# In vitro characterization of jellyfish venom fibrin(ogen)olytic enzymes from *Nemopilema nomurai*

**DOI:** 10.1186/s40409-017-0125-8

**Published:** 2017-07-19

**Authors:** Seong Kyeong Bae, Hyunkyoung Lee, Yunwi Heo, Min Jung Pyo, Indu Choudhary, Chang Hoon Han, Won Duk Yoon, Changkeun Kang, Euikyung Kim

**Affiliations:** 1 College of Veterinary Medicine, Gyeongsang National University, Jinju, 660-701 Korea; 2 Headquarters for Marine Environment, National Fisheries Research & Development Institute, Shiran-ri, Gijang-eup, Gijang-gun, Busan, 619-705 Korea; 3 Gyeongnam Department of Environment & Toxicology, Korea Institute of Toxicology, Gyeongnam 52834, Jinju, Korea

**Keywords:** *Nemopilema nomurai*, Jellyfish venom, Chymotrypsin, Serine protease, Fibrinolytic activity

## Abstract

**Background:**

Because jellyfish are capable of provoking envenomation in humans, they are considered hazardous organisms. Although the effects of their toxins are a matter of concern, information on the venom components, biological activity and pathological mechanisms are still scarce. Therefore, the aim of the present study was to investigate a serine protease component of *Nemopilema nomurai* jellyfish venom (NnV) and unveil its characteristics.

**Methods:**

To determine the relationship between fibrinolytic activity of NnV and the serine protease, fibrin zymography was performed using metalloprotease and serine protease inhibitors. The biochemical characterization of serine proteases of NnV were determined by the amidolytic assay. Fractions with fibrinolytic activity were obtained by DEAE cation exchange column.

**Results:**

NnV displayed fibrinolytic activities with molecular masses of approximately 70, 35, 30, and 28 kDa. The fibrinolytic activity of NnV was completely obliterated by phenylmethylsulfonyl fluoride, a prototype serine protease inhibitor. Based on amidolytic assays using chromogenic substrates specific for various kinds of serine proteases, NnV predominantly manifested a chymotrypsin-like feature. Its activity was completely eliminated at low pH (< 6) and high temperatures (> 37 °C). Some metal ions (Co^2+^, Cu^2+^, Zn^2+^ and Ni^2+^) strongly suppressed its fibrinolytic activity, while others (Ca^2+^ and Mg^2+^) failed to do so. Isolation of a serine protease with fibrionolytic activity from NnV revealed that only p3 showed the fibrinolytic activity, which was completely inhibited by PMSF.

**Conclusion:**

The present study showed that *N. nomurai* jellyfish venom has a chymotrypsin-like serine protease with fibrinolytic activity. Such information might be useful for developing clinical management of jellyfish envenomation and pharmacological agents with therapeutic potential for thrombotic diseases in the future.

## Background

Over the last decade, there has been a dramatic increase of global jellyfish blooming from many places in the world, including the oceans of Korea, China, and Japan. Jellyfish blooming can cause a number of social, economic, and public health problems. Power plants may shutdown due to jellyfish clogging the cooling water system. In addition, jellyfish can damage fishery industries and sting humans [1–3]. Jellyfish stinging is considered a serious envenomation for sea bathers due to its life-threating effects, including diffused neurotoxicity, cardiovascular collapse, respiratory failure, hypotension, shock and even death [4–7]. However, only a few toxic components have been successfully identified from jellyfish venom. Their molecular mechanisms of actions remain unclear from toxicological/pathological point of view.

*Nemopilema nomurai* is a giant jellyfish with a bell size up to 2 m in diameter. This jellyfish is one of the dominant jellyfish species in Korean coast. Its sting accidents have been increasing every year [8]. *N. nomurai* venom causes various symptoms that are mild or severe such as itching, redness, edema, hypotension, shock, and even death [9]. To date, several reports revealed that NnV induces cardiotoxic, cytotoxic, dermonecrotic, hemolytic, myotoxic, and proteolytic effects in in vitro and in vivo studies [10–13]. Our previous study has demonstrated that NnV has a proteolytic activity which is closely associated with cytotoxicity in skin cells [13]. Co-treatment of NnV with 1, 10-phenanthroline (metalloprotease inhibitor) can suppress its proteolytic activity on gelatin, fibrin, and casein as well as its cytotoxicity [13]. Furthermore, it has been demonstrated that the metalloprotease activity of NnV plays an important role in dermal pathology by its envenomation [12]. NnV causes severe damage accompanied by collapse of skin barriers, hemorrhage, and neutrophil infiltration in dermis. Treatment with tetracycline (a metalloprotease inhibitor) can alleviate pathological skin lesion. Therefore, like snake venom, the proteolytic activity might play a central role in local pathological alterations caused by *N. nomurai* sting.

The proteolytic activity of venom is mainly associated with two protease groups: metalloprotease and serine protease. Serine protease is particularly abundant in snake venoms. It can directly affect the coagulation cascade [14]. It influences the degradation of coagulation factor, disturbance of platelet aggregation, and fibrinolysis, thus preventing clot formation and causing systemic bleeding, hypovolemia, and hemodynamic shock [14, 15]. Serine protease also influences cell differentiation, immune response, and digestion [14, 16]. Transcriptomics and proteomics analysis have shown that serine protease is one toxic component in jellyfish venoms [17–19]. Our previous study has found a chymotrypsin-like serine protease in NnV and determined its full-length cDNA and gene sequence [20]. However, little information is available for serine protease in NnV. Therefore, the objective of this study was to investigate the serine protease component of NnV and unveil its characteristics using various biochemical methods.

## Methods

### Chemicals and reagents

Fibrinogen (type I-S from bovine plasma), thrombin (from bovine plasma), 1, 10-phenanthroline, phenylmethanesulfonyl fluoride (PMSF), benzamidine and chromogenic substrates were purchased from Sigma Chemical Co. (USA). Ethylenediaminetetraacetic acid (EDTA) and β-mercaptoethanol were obtained from Amresco Chemical (USA). All other reagents used were of the purest grade available.

### Jellyfish nematocyst preparation

Five different species of jellyfish samples were collected from various geographical locations around the coasts of South Korea as follows: *N. nomurai* jellyfish from the Korea Strait along the coasts of Geoje in September 2012; *Aurelia aurita* from Masan in September 2012; *Dactylometra quinquecirrha* from Tongyoung in August 2011; *Physalia physalis* from the Jeju island in July 2012; and *Carybdea brevipedalia* from the South sea near Samcheonpo in August 2012. Only tentacles were collected and transferred immediately to a laboratory for further preparation. Nematocysts were isolated from the dissected tentacles as described by the method of Bloom with slight modifications [21].

In brief, dissected tentacles were rinsed with cold seawater to remove debris. The tentacles were placed in three volumes of cold seawater for 24 h with gentle swirling for 1 h once a day at 4 °C. After autolysis for 24 h at 4 °C, the supernatant was collected and centrifuged at 4000 g for 10 min. The settled material was resuspended in fresh seawater and set for autolysis for 24 h. This process was repeated for three days. The sediments were collected and centrifuged 4000 g for 10 min and washed several times with fresh distilled water by centrifugation 100 g at 4 °C for 5 min until debris around nematocysts was almost removed. Finally, the undischarged nematocysts were collected, lyophilized and stored at −70 °C until use.

### Venom preparation

Venom was extracted from the freeze-dried nematocysts using the technique described by Carrette and Seymour [22] with a minor modification. In brief, venom was extracted from 60 mg of nematocyst powder using glass beads (approximately 8000 beads; 0.5 mm in diameter) and 1 mL of cold phosphate buffered saline (PBS, pH 7.4). These mixtures were shaken in a mini bead mill at 3000 rpm (40 s) for ten times with intermittent cooling on ice. The venom extracts were then transferred to a new microfuge tube and centrifuged (13,000×g) at 4 °C for 30 min. The supernatant was used as venom in the present study. Protein concentration of the venom was determined by using Bradford method and the venom was employed based on its protein concentration [23].

### SDS polyacrylamide gel electrophoresis (SDS-PAGE)

Electrophoresis was carried out according to Laemmli method [24] using 12% separating gel and 4% stacking gel. The NnV was prepared in non-reducing sample buffer (4% SDS, 125 mM Tris-HCl, pH 6.8, 20% glycerol, 0.01% bromophenol blue) then stored at −20 °C until use. NnV protein (50 μg) was electrophoresed for 90 min at 100 V at constant voltages using Tris-glycine running buffer. The molecular weight markers, 10–250 kDa (Precision Plus Protein™ Standards, Bio-rad, USA) were run parallel with NnV for molecular weight estimation. Following electrophoresis, separated protein bands were stained with 0.125% Coomassie blue in 40% methanol, 10% acetic acid.

### Examination of fibrinolytic activity using fibrin zymography

Fibrin was used as substrate for the evaluation of fibrinolytic activity in zymography assay. For this experiment, fibrinogen (0.6 mg/mL) and thrombin (0.01 unit/mL) dissolved in 20 mM sodium phosphate buffer (pH 7.4) was copolymerized with 12% polyacrylamide to prepare the respective zymography gel. The various venoms (20 μg) to be analyzed were prepared in non-reducing sample buffer, then run on gels at 100 V at 4 °C. After electrophoresis, SDS was removed by washing the gel twice for 30 min in 2.5% Triton X-100. Then, the gel was incubated with 20 mM Tris (pH 7.4), 0.5 mM calcium chloride, 200 mM sodium chloride at 37 °C for 16 h and the gel was stained with 0.125% Coomassie blue. Clear zones of the gel indicate regions of fibrinolytic activity. To know the effect of metal ions and protease inhibitors on fibrinolytic activity, the metal ions and protease inhibitors were added to incubation buffers [20 mM Tris (pH 7.4), 200 mM sodium chloride], and the gel stained as usual. All metal ions and inhibitors give a final concentration of 2 mM except PMSF (1 mM).

### Fibrinogenolytic activity

Fibrinogenolytic activity of NnV was investigated according to the method by Matsubara et al. [25]. Briefly, 15 μL of bovine fibronogen (20 mg/mL) was incubated with 10 μL NnV and 5 μL of reaction buffer (pH 7.4, 200 mM sodium chloride, 0.5 mM calcium chloride, 20 mM Tris) at 37 °C for indicated times and doses. The digested products were analyzed using 7.5% SDS-PAGE.

### Amidolytic activity assay

In an attempt to evaluate cleavage specificity, the amidolytic activity of NnV was assessed using various chromogenic substrates: N-Succinyl-Ala-Ala-Ala-*ρ*NA (for elastase), Nα-Benzoyl-DL-Arg-*ρ*NA (for trypsin), N-(p-Tosyl)-Gly-Pro-Lys-4 nitroanilide acetate salt (for plasmin), N-Benzoyl-Phe-Val-Arg-*p*-nitroanilide hydrochloride (for thrombin), N-Succinyl-Ala-Ala-Pro-Phe-*ρ*NA (for chymotrypsin). For the assays, each substrate was made up to 0.5 mM and then incubated with NnV (0.1 mg/mL) for 1 h at 37 °C. The reactions were monitored every five minutes using PowerWave XS microreader (Biotek) at 405 nm.

### Effects of pH and temperature on amidolytic activity of NnV

The effect of pH on the enzymatic activity of NnV was evaluated under diverse pH conditions. NnV was incubated with different pH buffers, including 0.5 M acetate (pH 3 and 4), 0.1 M phosphate (pH 5, 6, 7 and 8) and 0.5 M glycine-NaOH (pH 9, 10 and 11) for 1 h at 4 °C. It reacted against N-Succinyl-Ala-Ala-Pro-Phe- *ρ*NA (for chymotrypsin) substrate and the proteolysis of the substrate was detected at 405 nm. Furthermore, the enzymatic activity was assessed at various temperature conditions at 4, 25, 37, 60 and 100 °C. The residual activity was measured after incubation of NnV in different temperatures for 30 min.

### Effects of metal ions and protease inhibitors on enzymatic activity of NnV

The substrate of N-Succinyl-Ala-Ala-Pro-Phe-*ρ*NA (for chymotrypsin) was used to measure the effects of metal ions and inhibitors on the enzymatic activity of NnV. For this assay, NnV was incubated with several divalent metal ions or protease inhibitors for 1 h and followed by reacting chymotrypsin substrate for 30 min at 37 °C.

### Isolation of fibrinolytic protease from *N. nomurai* venom

Crude venom of NnV (180 mg) was dissolved in 10 mM Tris-HCl buffer (pH 7.8) and centrifuged at 13000×*g* for 30 min. The venom solution was resolved on DEAE column (GE Healthcare), previously dialyzed against extraction buffer at 4 °C. The column was eluted with gradient concentration of NaCl from 0 to 0.8 M at a flow rate of 1 mL per min. Each peak was tested for fibrinolytic activity and SDS-PAGE.

### Statistical analysis

The results are expressed as mean ± standard deviation (SD). One-way analysis of variance (ANOVA) was used to evaluate the significance of difference between the two mean values. The values of *p* considered statistically significant were *p* < 0.01 and *p* < 0.05.

## Results

### SDS-PAGE and fibrinolytic activity of NnV

**Fig. 1:**
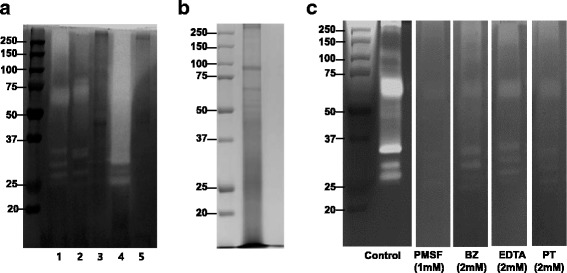
SDS-PAGE profile and fibrinolytic activity of NnV. **a** We compared the fibrinolytic activity between jellyfish species. Lane 1 – *N. nomurai*; lane 2 – *A. aurita*; lane 3 – *D. quinquecirrha*; lane 4 – *P. physalis*, lane 5 – *C. brevipedalia*. **b** NnV (50 μg) was submitted to SDS electrophoresis under non-reducing conditions. The gels were stained with 0.125% Coomassie blue. **c** Fibrin zymography of NnV with various protease inhibitors, including PMSF (1 mM), BZ (2 mM), EDTA (2 mM) and PT (2 mM). Clear zones in the fibrin gel indicated regions of proteolytic activity

To compare the fibrinolytic activity between different jellyfish species, their venom proteins were loaded into fibrin zymogrpahy. The fibrinolytic activity indicated venom of three species, namely *N. nomurai*, *A. aurita* and *P. physalis*, but not *D. quinquecirrha* or *C. brevipedalia*. Among these, *P. physalis* venom was highly potent for fibrin degradation and its molecular weight was above 25 kDa. *N. nomurai* and *A. aurita* venoms showed similar banding patterns, which were distributed in 60–80 kDa and 25–37 kDa (Fig. 1a). The 90 and 70 kDa bands showed more intense staining than other bands.

In fibrin zymogrpahy, strong fibrinolytic activity was correlated with a molecular weight of approximately 70 kDa, while weaker hydrolytic activity was found for the three bands with molecular masses between 37 and 25 kDa (Fig. 1b). Fibrinolytic enzymes were categorized into two groups (metalloprotease and serine protease) according to their action mechanisms and site. To determine which kind of protease was associated with the fibrionolytic activity of NnV, broad inhibitors for metalloprotease [EDTA and 1, 10-Phenanthroline (PT)] and serine protease [PMSF and benzamidine (BZ)] were incubated with NnV. Results showed that all inhibitors partially inhibited the fibrinolytic activity of NnV. However, only PMSF at a low concentration of 1 mM showed strong inhibitory effect on its fibrinolytic activity (Fig. 1c). These results suggest that serine proteases play a more important role in the fibrinolytic activity of NnV than metallorproteases.

### Fibrinogenolytic activity of NnV

**Fig. 2:**
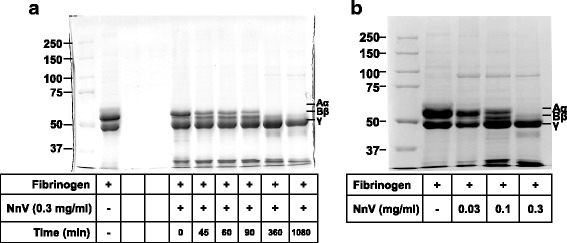
Fibrinogenolytic activity of NnV. NnV was incubated with fibrinogen (20 mg/mL) for the indicated (**a**) times and (**b**) doses at 37 °C. The mixture samples were electrophoresed in the 7.5% SDS-PAGE and stained with Coomassie blue. Fibrinogen consists of three polypeptides chains α, β and γ

The fibrinogenolytic activity of NnV was analyzed by SDS-PAGE. NnV degraded both α-chain and β-chain of fibrinogen in dose- and time-dependent manners (Fig. 2a and b). The α-chain of fibrinogen was immediately digested after incubation with NnV. Its complete degradation was observed at 360 min after incubation with NnV. The β-chain of fibrinogen began to be degraded at 360 min after incubation with NnV. However, γ-chain of fibrinogen was not affect by NnV.

### Amidolytic activity of NnV

**Fig. 3:**
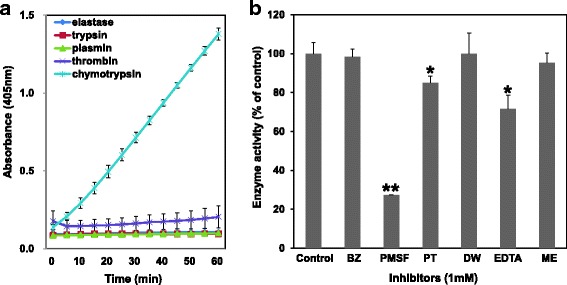
The catalytic activity of NnV on several chromogenic substrates. **a** NnV was preincubated with different substrates [N-Succinyl-Ala-Ala-Ala-*ρ*NA (for elastase), Nα-Benzoyl-DL-Arg-*ρ*NA (for trypsin), N-(p-Tosyl)-Gly-Pro-Lys-*ρ*NA·acetate salt (for plasmin), N-Benzoyl-Phe-Val-Arg-*ρ*NA·HCl (for thrombin), N-Succinyl-Ala-Ala-Pro-Phe-*ρ*NA (for chymotrypsin)], 0.5 mM for 1 h. The catalytic activity of NnV was assayed at 405 nm. **b** NnV was preincubated with different protease inhibitors (1 mM), followed by reacting chymotrypsin substrate for 30 min. Data represent as mean ± SD from the three fields *p < 0.05 and **p < 0.01, compared to control

NnV failed to degrade elastase, trypsin, thrombin, and plasmin substrates. However, it specifically exhibited hydrolytic activity against N-Succinyl-Ala-Ala-Pro-Phe-*ρ*NA substrate (for chymotrypsin) (Fig. 3a). In addition, such enzymatic activity of NnV on chymotrypsin substrate was strongly inhibited by PMSF (Fig. 3b).

### Hydrolytic activity of NnV is dependent on pH, temperature, metal ions, and protease inhibitors

**Fig. 4:**
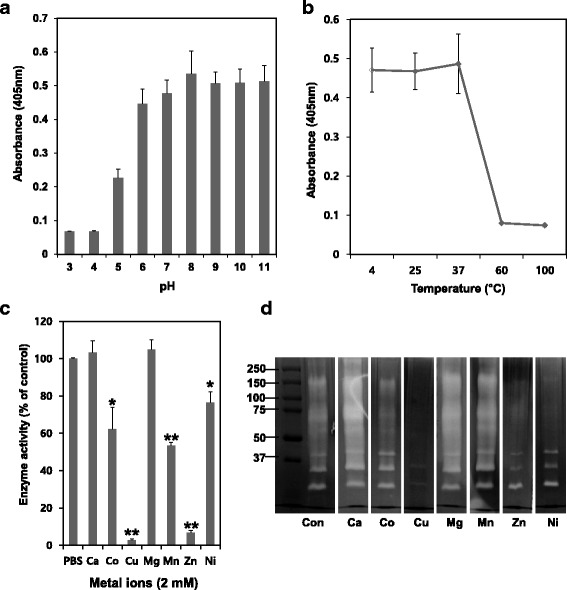
The catalytic activity of NnV was dependent on pH, temperature and metal ions. NnV (2 mg/mL) was incubated under different (**a**) pH (3, 4, 5, 6, 7, 8, 9, 10 and 11), (**b**) temperatures (4, 25, 37, 60 and 100 °C) and (**c**) metal ions (Ca^2+^, Co^2+^, Cu^2+^, Mg^2+^, Mn^2+^, Zn^2+^ and Ni^2+^) and added to chymotrypsin substrate for 30 min at 37 °C. **d** NnV (20 μg) was submitted to fibrin zymography and incubated with metal ions (10 mM) in zymography reaction buffer for 18 h at 37 °C. The fibrin gel was stained with 0.125% Coomassie blue. Data represent as mean ± SD from the three fields *p < 0.05 and **p < 0.01, compared to control

The effect of pH on the enzymatic activity of NnV was determined using buffers at various pH values. The enzymatic activity of NnV on chymotrypsin substrate was stable and higher at neutral (pH 7) and basic (pH 8, 9, 10 and 11) pH conditions (Fig. 4a). However, its activity was unstable and lost under acidic conditions. The optimum temperature condition for its activity was found to be between 4 and 37 °C. However, its activity was lost at high temperature including 60 and 100 °C (Fig. 4b). The effects of various metal ions on its enzymatic activity were also examined. Ca^2+^ and Mg^2+^ failed to activate or inhibit its enzymatic activity and fibrinolytic activity. However, Co^2+^, Mn^2+^, and Ni^2+^ showed slightly inhibitory effect on its enzymatic and fibrinolytic activity (Fig. 4c and d). Particularly, Cu^2+^ and Zn^2+^ exerted completely inhibitory effects on its activities.

### Purification of NnV with fibrinolytic activity

**Fig. 5:**
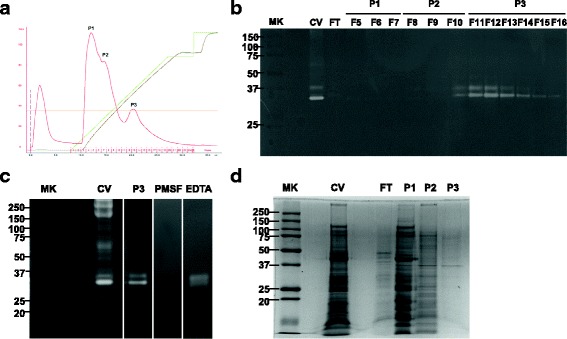
The purification of serine protease from NnV. **a** Crude venom was dissolved in 10 mM Tris-HCl (pH 7.8) buffer and centrifuged for 30 min at 13000 × g. The supernatant was loaded on a DEAE column and the proteins were eluted with a NaCl linear gradient of 0 to 80% at flow rate of 1 mL/min. **b** and **d** The fibrinolytic activity and protein profile of each fraction were evaluated by by zymography and SDS-PAGE. **c** The fibrinolytic activity was inhibited by PMSF 1 mM, but not by EDTA

After separating NnV on DEAE column, three peaks were obtained and the fibrionolytic activity of each peak was evaluated using fibrin zymography. Peak 3 (P3, Fr 11–15) showed fibrinolytic activity with two bands, whereas P1 and P2 failed to show any band (Fig. 5b). To determine whether the fibrinolytic activity of P3 was associated with serine protease, fibrin zymography was performed using typical protease inhibitors EDTA and PMSF. It was found that PMSF abolished the fibrinolytic activity of P3, but EDTA did not (Fig. 5c). However, the protein amount of P3 was small and the matching band with fibrinolytic activity at 35 kDa in P3 was very weak (Fig. 5d).

## Discussion

Several reports have demonstrated that jellyfish venom consists of various bioactive substances, including cytolysins, neurotoxins, peptides and proteases [13, 20, 26–28]. Unlike other venoms (snake, scorpion and spider), jellyfish venom is not secreted from venom glands or injected through specialized nematocysts that are present all over the cnidarians. Hence, it is very difficult to obtain pure jellyfish venom due to poor yield, besides being a time-consuming and technically challenging process [29]. Although it is not easy to isolate and characterize individual jellyfish venom components, their characterization is very important in various aspects, including clinical management, development of therapeutic agents, and pharmaceutical application.

Our previous study has revealed that NnV contains abundant proteolytic enzymes responsible for dermonecrosis and cytotoxicity after stings [12, 13]. Among proteolytic activities, fibrinolytic activity is an established mediator of toxicity in various animal venoms, especially snake venoms. Fibrinolytic enzymes may interfere with coagulation and fibrin(ogen)olytic systems, leading to systemic bleeding, coagulopathy, hypovolemia, and hemodynamic shock [14, 15, 30]. In our previous study, we reported the fibrinolytic activity of NnV, but it only 1,10-phenathroline was used as an enzymatic inhibitor. Therefore, the relationship between fibrinolytic activity and other proteases could not be excluded. Generally, proteases with fibrinolytic activity are closely associated with metalloproteases and serine proteases.

In order to determine which protease contributed to the fibrionolytic activity of NnV, in this study fibrin zymography was performed using metalloprotease inhibitors (EDTA and PT) and serine protease inhibitors (PMSF and BZ). Although EDTA and PT moderately inhibited the fibrinolytic activity, only PMSF completely inhibited it at a low concentration of 1 mM. Regarding its amidolytic activity, NnV only cleaved chymotrypsin substrates. Such activity was mostly inhibited by PMSF. Hence, we concluded that the fibrinolytic activity of NnV is mostly due to its serine proteases rather than metalloproteases. It demonstrated specific activity toward the chymotrypsin substrate.

The fibrinolytic activity of NnV was dependent on pH, temperature, and metal ion concentration. Its fibrinolytic activity was strong at pH values of 8 to 11. However, such activity was lost at pH values of 3 to 5 and weakened at pH values of 6 to 7. To date, fibrinolytic enzymes derived from venoms have been found to be stable at pH values of 5.5 to 8.5. For example, the fibrinolytic activity of neuwiedase, a metalloprotease from *Bothrops neuwiedi* snake, has been found to be strong at a pH range of 7.4 to 8.0 and Brevilysin L6 can persevere its activity at optimal pH of 8.5 to 9.5 [31, 32]. Interestingly, fibrinolytic activity of NnV was found to be stable at basic conditions in this study, even at pH 11. Its activity was fast at temperature below 37 °C. However, this activity was sharply reduced when temperature was increased to be above 37 °C. Furthermore, the fibrinolytic activity of NnV was strongly inhibited by Zn^2+^ and Cu^2+^ at 1 mM in fibrin zymography and chromogenic substrate test.

Fibrinolytic enzymes commonly have fibrinogenolytic activities. Therefore, we performed assays to evaluate the fibrinogenolytic activity of NnV and found that NnV rapidly degraded α-chain of fibrinogen followed by β-chain in a dose- and time-dependent manner. However, NnV failed to degrade γ-chain of fibrinogen. Most serine proteases in snake venoms preferentially hydrolyze β-chain of fibrin(ogen) with low activity toward α-chain of fibrin(ogen). However, there are a few exceptions to such characteristic feature. For example, Dnase purified from the venom of *Deinagkistrodon acutus* is found to be a fibrinogenase that belongs to the serine protease family which preferentially digests α-chain of fibrinogen in comparison with β-chain [33]. Despite the fact that fibrinogenase isolated from *Agkistrodon halys* brevicaudus is a metalloprotease, it can rapidly cleave β-chain of fibrinogen [32]. Although the fibrin(ogen)olytic enzymes of NnV belonged to the serine protease group, it preferentially degraded α-chain than β-chain of fibrinogen.

To isolate fibrinolytic enzymes from NnV, we performed chromatography on DEAE column. Among three peaks, only P3 showed fibrinolytic activity with molecular weight of approximately 35 and 37 kDa. Its fibrinolytic activity was completely inhibited by PMSF, but not by EDTA, indicating that the fibrinolytic enzyme in P3 belongs to the serine protease family. Despite the fibrinolytic activity of P3, its protein profile was very week. Hence, it is difficult to identify or characterize its active sources. Obtaining sufficient amounts of NnV for identification and characterization remains a significant challenge.

Serine proteases in venoms are associated with various biological activities, including hemostatic, cell differentiation, prey digestion as well as affecting the complement system [30]. It mainly disturbs the coagulation cascade through activation or inactivation of platelet aggregation, coagulation, and fibrinolysis [15]. Coagulopathy has not yet been observed in patients stung by jellyfish. Therefore, we speculate the reasons why serine proteases are present in jellyfish venom. Serine proteases can act as spreading factors that may increase their permeability into tissue and promote the spread of venom. They can also form complexes capable of activating the complement cascade through the cleavage of specific components, leading to induction and facilitation of inflammation [34, 35]. Although this study did not investigate the pathological mechanisms of serine proteases, it could explain that inflammatory symptoms provoked by jellyfish envenomation may be partially caused by serine proteases.

## Conclusions

Jellyfish venom research is a very attractive field due to increasing sting accidents and the presence of various bioactive components. Biochemical characterization of toxins can help us understand the pathological symptoms associated with envenomation, so that clinical agents can be developed. Although the present study partially purified a serine protease from NnV, our results could aid the development of clinical management. Furthermore, fibrinolytic properties can be used to treat thrombosis by preventing clot formation [36, 37]. Based on the results of this study, further research can be conducted to isolate serine proteases and investigate the pathological mechanisms involved in inflammatory reactions, in addition to their thrombolytic potential.

## Abbreviations

NnV: *Nemopilema nomurai* jellyfish venom
PMSF: Phenylmethanesulfonyl fluoride
SD: Standard deviation
SDS-PAGE: SDS polyacrylamide gel electrophoresis
